# Crystal structure of β-benzyl dl-aspartate *N*-carb­oxyanhydride

**DOI:** 10.1107/S2056989017003024

**Published:** 2017-02-24

**Authors:** Hitoshi Kanazawa, Aya Inada

**Affiliations:** a Faculty of Symbiotic Systems Science, Fukushima University, 1 Kanayagawa, Fukushima, 960-1296, Japan

**Keywords:** crystal structure, solid state polymerization, amino acid, *N*-carb­oxyanhydride, hydrogen bonding

## Abstract

In the title racemic compound, C_12_H_11_NO_5_ [systematic name: benzyl 2-(2,5-dioxooxazolidin-4-yl)acetate], the oxazolidine ring is planar, with an r.m.s. deviation of 0.03 Å. The benzyl ring is almost normal to the oxazolidine ring, making a dihedral angle of 80.11 (12)°. In the crystal, inversion dimers are formed between the l- and d-enanti­omers *via* pairs of N—H⋯O hydrogen bonds. This arrangement is favourable for the polymerization of the compound in the solid state. The dimers are linked by C—H⋯O hydrogen bonds, forming layers parallel to the *ab* plane.

## Abstract 2

In the title racemic compound, the benzyl ring is almost normal to the oxazolidine ring, with a dihedral angle of 80.11 (12)°. In the crystal, inversion dimers are formed between the l- and d-enanti­omers *via* pairs of N—H⋯O hydrogen bonds.

## Chemical context   

*N*-Carb­oxyanhydrides (NCAs) of amino acids are used ex­tensively as monomers for the preparation of high mol­ecular weight polypeptides (Kricheldorf, 2006 ▸). Amino acid NCAs are easily soluble but the resulting polypeptides are not soluble in general organic solvents. Only a few amino acid ester NCAs such as γ-benzyl l-glutamate NCA and γ-benzyl l-aspartate NCA are polymerized in solutions, because the resulting polypeptides are soluble in them. Thus, the polymerization of these amino acid ester NCAs has been investigated by many researchers. On the other hand, we found that every amino acid NCA crystal is polymerized in the solid state in hexane by the initiation of amines, and we have studied the solid-state polymerization of amino acid NCAs with reference to their crystal structures (Kanazawa, 1992 ▸, 1998 ▸; Kanazawa *et al.*, 1978 ▸, 2006 ▸). We have studied the polymerization of γ-benzyl l-aspartate NCA (BLA NCA) initiated by butyl amine in solution and the solid state (Kanazawa & Sato, 1996 ▸), and determined the crystal structure of BLA NCA (Kanazawa & Magoshi, 2003 ▸), to consider the high reactivity in the solid state. In addition, we have attempted the preparation of single crystals of the title compound, β-benzyl dl-aspartate NCA (BDLA NCA). The BDLA NCA single crystals were obtained by a slow crystallization in solutions. The polymerization of BDLA NCA was carried out both in dioxane solution and in the solid state in hexane, using butyl amine as initiator. BDLA NCA is not so reactive in solutions; the existence of l- and d-enanti­omers in solution seems unfavourable for fast polymerization. On the other hand, the compound is very reactive in the solid state. It is therefore important to determine its crystal structure in order to consider the difference in the reactivity between the solution and the solid state.
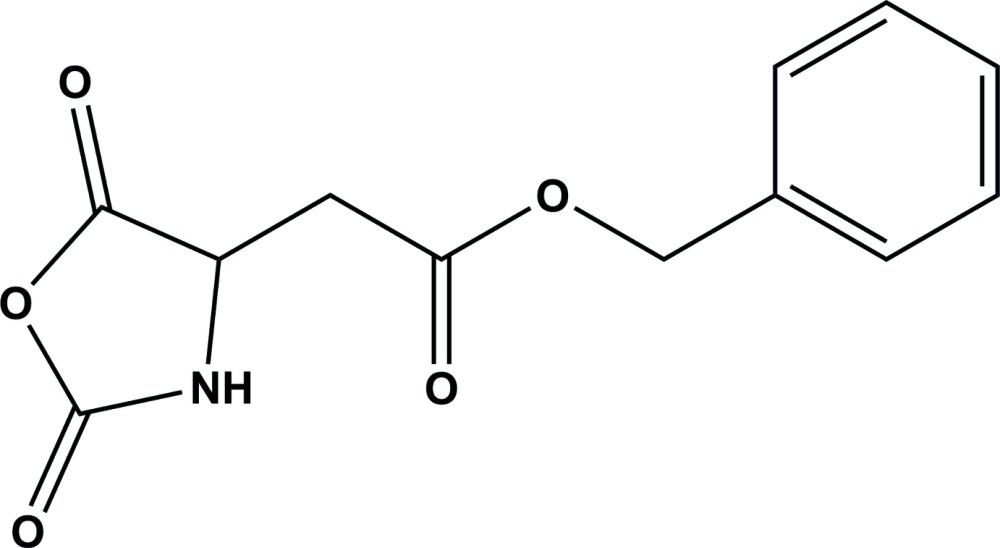


## Structural commentary   

**Figure 1:**
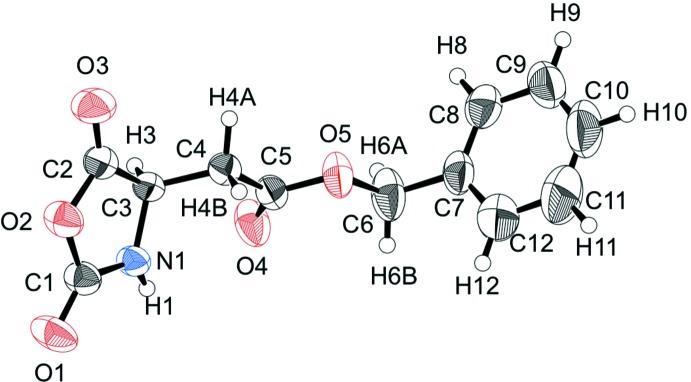
The mol­ecular structure of the title compound, showing the atom labelling and 50% probability displacement ellipsoids.

The mol­ecular structure of the title compound is shown in Fig. 1 ▸. The oxazolidine ring is planar, with a maximum deviation of 0.027 (2)Å for atom C1. The side chain has an extended conformation with the torsion angles C3—C4—C5—O5 and C4—C5—O5—C6 being 178.29 (14) and −179.29 (17)°, respectively. The benzyl ring is almost normal to the oxazolidine ring, making a dihedral angle of 80.11 (12)°.

## Supra­molecular features   

**Figure 2:**
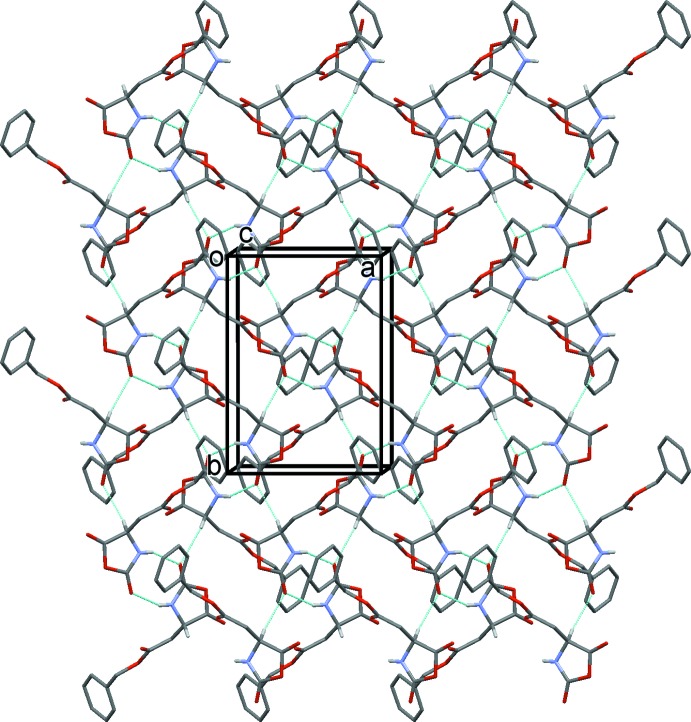
Crystal packing of the title compound, viewed along the *c* axis, showing the hydrogen bonds as dashed lines (see Table 1 ▸).

In the crystal, β-benzyl l-aspartate NCA and β-benzyl d-aspartate NCA mol­ecules form a dimer structure around a crystallographic center of symmetry *via* a pair of N1—H1⋯O1^i^ hydrogen bonds (Fig. 2 ▸ and Table 1 ▸). The dimers are linked by C—H⋯O hydrogen bonds, forming layers parallel to the *ab* plane (Fig. 2 ▸ and Table 1 ▸). The five-membered oxazolidine rings are packed in a layer and the –CH_2_COOCH_2_C_6_H_5_ groups are packed in another layer; these two different layers are stacked alternately. This sandwich structure is one of the important requirements for high reactivity in the solid state, because the five-membered rings can react with each other in the layer.

## Synthesis and crystallization   

The synthesis of BDLA was carried out by the reaction of dl-aspartic acid with benzyl alcohol in a manner similar to that for γ-benzyl l-glutamate (BLG) (Kanazawa, 1992 ▸). The title compound was obtained by the reaction of BDLA with triphosgene in tetra­hydro­furan, as reported previously for BLA NCA (Kanazawa & Magoshi, 2003 ▸). The reaction product was recrystallized slowly in a mixture of ethyl acetate and hexane (1:50 *v*/*v*), avoiding moisture contamination, giving colourless prismatic crystals.

## Refinement   

Crystal data, data collection and structure refinement details are summarized in Table 2 ▸. The N-bound H atom was located in a difference Fourier map and refined with *U*_iso_(H) = 1.2*U*_eq_(N). The C-bound H atoms were positioned geometrically (C—H = 0.93–0.98 Å) and treated as riding with *U*_iso_(H) = 1.2*U*_eq_(C).

## Supplementary Material

Crystal structure: contains datablock(s) I. DOI: 10.1107/S2056989017003024/su5349sup1.cif

Structure factors: contains datablock(s) I. DOI: 10.1107/S2056989017003024/su5349Isup2.hkl

Supporting information file. DOI: 10.1107/S2056989017003024/su5349Isup3.cml

CCDC reference: 1534297

Additional supporting information:  crystallographic information; 3D view; checkCIF report

## Figures and Tables

**Table 1:** Hydrogen-bond geometry (Å, °)

*D*—H⋯*A*	*D*—H	H⋯*A*	*D*⋯*A*	*D*—H⋯*A*
N1—H1⋯O1^i^	0.83 (2)	2.13 (2)	2.913 (3)	157 (2)
C3—H3⋯O1^ii^	0.98	2.39	3.101 (2)	129

Symmetry codes: (i) 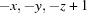; (ii) 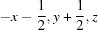.

**Table 2:** Experimental details

Crystal data	
Chemical formula	C_12_H_11_NO_5_
*M*_r_	249.22
Crystal system, space group	Orthorhombic, *P* *b* *c* *a*
Temperature (K)	293
*a*, *b*, *c* (Å)	8.6065 (8), 12.1558 (12), 23.820 (2)
*V* (Å^3^)	2492.0 (4)
*Z*	8
Radiation type	Mo *K*α
μ (mm^−1^)	0.11
Crystal size (mm)	0.43 × 0.23 × 0.03

Data collection	
Diffractometer	Rigaku XtaLAB mini
Absorption correction	Multi-scan (*REQAB*; Rigaku, 1998 ▸)
*T*_min_, *T*_max_	0.862, 0.997
No. of measured, independent and observed [*I* > 2σ(*I*)] reflections	24433, 2861, 1520
*R*_int_	0.084
(sin θ/λ)_max_ (Å^−1^)	0.649

Refinement	
*R*[*F* ^2^ > 2σ(*F* ^2^)], *wR*(*F* ^2^), *S*	0.047, 0.115, 0.98
No. of reflections	2861
No. of parameters	166
H-atom treatment	H atoms treated by a mixture of independent and constrained refinement
Δρ_max_, Δρ_min_ (e Å^−3^)	0.13, −0.16

Computer programs: *CrystalClear* (Rigaku, 2009 ▸), *SHELXS97* and *SHELXL97* (Sheldrick, 2008 ▸), *CrystalStructure* (Rigaku, 2009 ▸) and *Mercury* (Macrae *et al.*, 2008 ▸).

## supplementary crystallographic information

### Crystal data

**Table:**

C_12_H_11_NO_5_	*F*(000) = 1040
*M**_r_* = 249.22	*D*_x_ = 1.328 Mg m^−^^3^
Orthorhombic, *P**b**c**a*	Mo *K*α radiation, λ = 0.71075 Å
Hall symbol: -P 2ac 2ab	Cell parameters from 15837 reflections
*a* = 8.6065 (8) Å	θ = 3.0–27.6°
*b* = 12.1558 (12) Å	µ = 0.11 mm^−^^1^
*c* = 23.820 (2) Å	*T* = 293 K
*V* = 2492.0 (4) Å^3^	Prism, colourless
*Z* = 8	0.43 × 0.23 × 0.03 mm

### Data collection

**Table:**

Rigaku XtaLAB mini diffractometer	2861 independent reflections
Radiation source: fine-focus sealed tube	1520 reflections with *I* > 2σ(*I*)
Graphite monochromator	*R*_int_ = 0.084
Detector resolution: 6.849 pixels mm^-1^	θ_max_ = 27.5°, θ_min_ = 3.0°
ω scans	*h* = −11→11
Absorption correction: multi-scan (*REQAB*; Rigaku, 1998)	*k* = −15→15
*T*_min_ = 0.862, *T*_max_ = 0.997	*l* = −30→30
24433 measured reflections	

### Refinement

**Table:**

Refinement on *F*^2^	Primary atom site location: structure-invariant direct methods
Least-squares matrix: full	Secondary atom site location: difference Fourier map
*R*[*F*^2^ > 2σ(*F*^2^)] = 0.047	Hydrogen site location: inferred from neighbouring sites
*wR*(*F*^2^) = 0.115	H atoms treated by a mixture of independent and constrained refinement
*S* = 0.98	*w* = 1/[σ^2^(*F*_o_^2^) + (0.0503*P*)^2^] where *P* = (*F*_o_^2^ + 2*F*_c_^2^)/3
2861 reflections	(Δ/σ)_max_ = 0.020
166 parameters	Δρ_max_ = 0.13 e Å^−^^3^
0 restraints	Δρ_min_ = −0.16 e Å^−^^3^

### Special details

**Table:**

Geometry. All esds (except the esd in the dihedral angle between two l.s. planes) are estimated using the full covariance matrix. The cell esds are taken into account individually in the estimation of esds in distances, angles and torsion angles; correlations between esds in cell parameters are only used when they are defined by crystal symmetry. An approximate (isotropic) treatment of cell esds is used for estimating esds involving l.s. planes.
Refinement. Refinement of F^2^ against ALL reflections. The weighted R-factor wR and goodness of fit S are based on F^2^, conventional R-factors R are based on F, with F set to zero for negative F^2^. The threshold expression of F^2^ > 2sigma(F^2^) is used only for calculating R-factors(gt) etc. and is not relevant to the choice of reflections for refinement. R-factors based on F^2^ are statistically about twice as large as those based on F, and R- factors based on ALL data will be even larger.

### Fractional atomic coordinates and isotropic or equivalent isotropic displacement parameters (Å^2^)

**Table:**

	*x*	*y*	*z*	*U*_iso_*/*U*_eq_	
O1	−0.15716 (18)	−0.06365 (9)	0.53169 (7)	0.0695 (5)	
O2	−0.32140 (15)	0.05348 (9)	0.57507 (6)	0.0558 (4)	
O3	−0.43399 (16)	0.20638 (11)	0.60784 (7)	0.0696 (5)	
O4	0.09214 (16)	0.32273 (12)	0.53621 (6)	0.0675 (5)	
O5	0.12154 (15)	0.39128 (11)	0.62231 (6)	0.0592 (4)	
N1	−0.10097 (17)	0.11990 (11)	0.54204 (7)	0.0428 (4)	
C1	−0.1845 (2)	0.02906 (14)	0.54691 (8)	0.0475 (5)	
C2	−0.3277 (2)	0.16511 (14)	0.58457 (8)	0.0476 (5)	
C3	−0.1816 (2)	0.21622 (12)	0.56219 (7)	0.0392 (4)	
H3	−0.2066	0.2647	0.5306	0.047*	
C4	−0.0952 (2)	0.28038 (14)	0.60680 (8)	0.0434 (5)	
H4A	−0.1628	0.3370	0.6219	0.052*	
H4B	−0.0670	0.2314	0.6373	0.052*	
C5	0.0486 (2)	0.33268 (14)	0.58341 (9)	0.0462 (5)	
C6	0.2629 (3)	0.44719 (19)	0.60384 (10)	0.0760 (7)	
H6A	0.2391	0.5005	0.5748	0.091*	
H6B	0.3369	0.3945	0.5890	0.091*	
C7	0.3277 (2)	0.50317 (18)	0.65424 (9)	0.0582 (6)	
C8	0.2818 (2)	0.60778 (18)	0.66805 (10)	0.0649 (6)	
H8	0.2141	0.6455	0.6445	0.078*	
C9	0.3345 (3)	0.6578 (2)	0.71624 (13)	0.0841 (8)	
H9	0.3019	0.7286	0.7253	0.101*	
C10	0.4331 (4)	0.6039 (3)	0.75019 (13)	0.1036 (10)	
H10	0.4679	0.6375	0.7829	0.124*	
C11	0.4824 (4)	0.5014 (3)	0.73727 (16)	0.1274 (12)	
H11	0.5516	0.4652	0.7609	0.153*	
C12	0.4298 (3)	0.4500 (2)	0.68874 (14)	0.1006 (10)	
H12	0.4640	0.3796	0.6798	0.121*	
H1	−0.023 (2)	0.1231 (14)	0.5219 (8)	0.051*	

### Atomic displacement parameters (Å^2^)

**Table:**

	*U*^11^	*U*^22^	*U*^33^	*U*^12^	*U*^13^	*U*^23^
O1	0.0806 (11)	0.0323 (7)	0.0957 (12)	−0.0054 (7)	0.0248 (9)	−0.0072 (7)
O2	0.0549 (9)	0.0408 (7)	0.0716 (10)	−0.0091 (6)	0.0160 (7)	−0.0024 (6)
O3	0.0517 (9)	0.0652 (9)	0.0918 (12)	0.0024 (7)	0.0223 (9)	−0.0117 (8)
O4	0.0644 (10)	0.0866 (11)	0.0514 (10)	−0.0233 (8)	0.0137 (8)	−0.0240 (8)
O5	0.0541 (8)	0.0741 (9)	0.0492 (9)	−0.0240 (7)	0.0029 (7)	−0.0147 (7)
N1	0.0395 (9)	0.0326 (8)	0.0562 (11)	−0.0019 (7)	0.0074 (8)	−0.0022 (7)
C1	0.0497 (12)	0.0366 (10)	0.0562 (14)	−0.0024 (9)	0.0075 (10)	0.0014 (9)
C2	0.0462 (12)	0.0437 (10)	0.0529 (12)	0.0010 (9)	0.0015 (10)	−0.0045 (9)
C3	0.0396 (10)	0.0329 (9)	0.0452 (11)	0.0028 (8)	0.0000 (9)	−0.0027 (7)
C4	0.0432 (11)	0.0428 (10)	0.0441 (11)	0.0005 (8)	0.0016 (9)	−0.0044 (8)
C5	0.0461 (11)	0.0462 (10)	0.0463 (12)	−0.0027 (9)	−0.0010 (10)	−0.0087 (10)
C6	0.0619 (14)	0.1002 (17)	0.0660 (16)	−0.0381 (13)	0.0104 (13)	−0.0191 (13)
C7	0.0450 (12)	0.0712 (14)	0.0583 (15)	−0.0193 (11)	0.0005 (11)	−0.0107 (11)
C8	0.0487 (13)	0.0744 (14)	0.0714 (16)	−0.0113 (11)	0.0008 (12)	0.0003 (12)
C9	0.0662 (17)	0.0880 (17)	0.098 (2)	−0.0203 (14)	0.0095 (16)	−0.0349 (16)
C10	0.085 (2)	0.144 (3)	0.082 (2)	−0.022 (2)	−0.0167 (17)	−0.041 (2)
C11	0.113 (3)	0.141 (3)	0.128 (3)	0.009 (2)	−0.070 (2)	−0.019 (2)
C12	0.089 (2)	0.0844 (18)	0.128 (3)	0.0070 (15)	−0.035 (2)	−0.0211 (18)

### Geometric parameters (Å, º)

**Table:**

O1—C1	1.2070 (19)	C4—H4B	0.9700
O2—C2	1.377 (2)	C6—C7	1.488 (3)
O2—C1	1.388 (2)	C6—H6A	0.9700
O3—C2	1.181 (2)	C6—H6B	0.9700
O4—C5	1.191 (2)	C7—C12	1.365 (3)
O5—C5	1.327 (2)	C7—C8	1.372 (3)
O5—C6	1.461 (2)	C8—C9	1.376 (3)
N1—C1	1.323 (2)	C8—H8	0.9300
N1—C3	1.443 (2)	C9—C10	1.343 (4)
N1—H1	0.827 (19)	C9—H9	0.9300
C2—C3	1.501 (2)	C10—C11	1.352 (4)
C3—C4	1.513 (2)	C10—H10	0.9300
C3—H3	0.9800	C11—C12	1.390 (4)
C4—C5	1.499 (2)	C11—H11	0.9300
C4—H4A	0.9700	C12—H12	0.9300
			
C2—O2—C1	108.89 (14)	O5—C5—C4	111.02 (17)
C5—O5—C6	115.67 (15)	O5—C6—C7	106.37 (17)
C1—N1—C3	112.76 (15)	O5—C6—H6A	110.5
C1—N1—H1	122.1 (12)	C7—C6—H6A	110.5
C3—N1—H1	123.1 (12)	O5—C6—H6B	110.5
O1—C1—N1	130.31 (19)	C7—C6—H6B	110.5
O1—C1—O2	120.71 (16)	H6A—C6—H6B	108.6
N1—C1—O2	108.98 (14)	C12—C7—C8	118.7 (2)
O3—C2—O2	121.75 (18)	C12—C7—C6	120.7 (2)
O3—C2—C3	129.78 (16)	C8—C7—C6	120.6 (2)
O2—C2—C3	108.46 (15)	C7—C8—C9	121.0 (2)
N1—C3—C2	100.67 (13)	C7—C8—H8	119.5
N1—C3—C4	114.58 (15)	C9—C8—H8	119.5
C2—C3—C4	112.05 (15)	C10—C9—C8	119.7 (3)
N1—C3—H3	109.7	C10—C9—H9	120.2
C2—C3—H3	109.7	C8—C9—H9	120.2
C4—C3—H3	109.7	C9—C10—C11	120.7 (3)
C5—C4—C3	111.29 (16)	C9—C10—H10	119.6
C5—C4—H4A	109.4	C11—C10—H10	119.6
C3—C4—H4A	109.4	C10—C11—C12	120.1 (3)
C5—C4—H4B	109.4	C10—C11—H11	120.0
C3—C4—H4B	109.4	C12—C11—H11	120.0
H4A—C4—H4B	108.0	C7—C12—C11	119.8 (3)
O4—C5—O5	124.41 (17)	C7—C12—H12	120.1
O4—C5—C4	124.57 (17)	C11—C12—H12	120.1
			
C3—N1—C1—O1	175.5 (2)	C6—O5—C5—C4	−179.29 (17)
C3—N1—C1—O2	−5.3 (2)	C3—C4—C5—O4	−1.9 (3)
C2—O2—C1—O1	−176.46 (19)	C3—C4—C5—O5	178.29 (14)
C2—O2—C1—N1	4.3 (2)	C5—O5—C6—C7	−177.72 (18)
C1—O2—C2—O3	179.69 (19)	O5—C6—C7—C12	89.3 (3)
C1—O2—C2—C3	−1.6 (2)	O5—C6—C7—C8	−88.3 (2)
C1—N1—C3—C2	4.1 (2)	C12—C7—C8—C9	−1.4 (3)
C1—N1—C3—C4	124.47 (18)	C6—C7—C8—C9	176.3 (2)
O3—C2—C3—N1	177.2 (2)	C7—C8—C9—C10	0.4 (4)
O2—C2—C3—N1	−1.29 (19)	C8—C9—C10—C11	0.6 (5)
O3—C2—C3—C4	55.0 (3)	C9—C10—C11—C12	−0.7 (6)
O2—C2—C3—C4	−123.50 (16)	C8—C7—C12—C11	1.3 (4)
N1—C3—C4—C5	67.26 (19)	C6—C7—C12—C11	−176.4 (3)
C2—C3—C4—C5	−178.86 (14)	C10—C11—C12—C7	−0.3 (5)
C6—O5—C5—O4	0.9 (3)		

### Hydrogen-bond geometry (Å, º)

**Table:**

*D*—H···*A*	*D*—H	H···*A*	*D*···*A*	*D*—H···*A*
N1—H1···O1^i^	0.83 (2)	2.13 (2)	2.913 (3)	157 (2)
C3—H3···O1^ii^	0.98	2.39	3.101 (2)	129

Symmetry codes: (i) −*x*, −*y*, −*z*+1; (ii) −*x*−1/2, *y*+1/2, *z*.
